# Mapping CushingQOL scores to EQ-5D utility values using data from the European Registry on Cushing’s syndrome (ERCUSYN)

**DOI:** 10.1007/s11136-013-0396-7

**Published:** 2013-03-29

**Authors:** X. Badia, M. Roset, E. Valassi, H. Franz, A. Forsythe, S. M. Webb

**Affiliations:** 1 Health Economics and Outcomes Research, IMS Health, C/ Dr. Ferran 25-27, 2º, 08034 Barcelona, Spain; 2 Endocrinology/Medicine Departments, Hospital Sant Pau, IIB-Sant Pau, Centro de Investigación Biomédica en Red de Enfermedades Raras (CIBERER Unit 747), ISCIII, Universitat Autònoma de Barcelona (UAB), Barcelona, Spain; 3 Lohmann & Birkner Health Care Consulting GmbH, Berlin, Germany; 4 Global Health Economics and Market Access, Novartis Oncology, East Hanover, NJ 07936-1080 USA

**Keywords:** Cushing’s syndrome, Mapping, Questionnaire, Quality of life, EQ-5D

## Abstract

**Purpose:**

To construct a model to predict preference-adjusted EuroQol 5D (EQ-5D) health utilities for CS using the disease-specific health-related quality of life measure (CushingQOL).

**Methods:**

Data were obtained from the European Registry on CS (ERCUSYN). ERCUSYN is a web-based, multicenter, observational study that enrolled 508 CS patients from 36 centers in 23 European countries. Patients included in the study completed both the EQ-5D and the disease-specific CushingQOL questionnaire. Socio-demographic and clinical data were also collected. The UK tariff values were used to calculate EQ-5D utility scores. Various predictive models were tested, and the final model was selected based on four criteria: explanatory power (adjusted R-squared), consistency of estimated coefficients (sign and parameter estimation), normality of prediction errors (mean error, mean absolute error, root mean squared error), and parsimony.

**Results:**

For the mapping analysis, data were available from a total of 129 patients. Mean (SD) age was 43.1 (13) years, and the sample was predominantly female (84.5 %). Patients had a mean (SD) CushingQOL score of 39.7 (17.1) and a mean (SD) ‘tariff’ value on the EQ-5D of 0.55 (0.3). The model which best met the criteria for selection included the intercept and 3 CushingQOL’s questions and had an R^2^ of 0.506 and a root mean square error of 0.216.

**Conclusions:**

It was possible to find a mapping function which successfully predicted the EQ-5D UK utilities from disease-specific CushingQOL scores. The function may be useful in calculating EQ-5D scores when EQ-5D data have not been gathered directly in a study.

## Introduction

Cushing’s syndrome (CS) is a rare hormonal disorder caused by chronic exposure to hypercortisolism, with an annual incidence of 2–3 cases per million [1]. It produces many symptoms and disorders including central obesity, gonadal dysfunction, hirsutism, delayed wound healing, muscle weakness, hypertension, hyperglycemia, osteoporosis, and depression, among others [2]. Psychiatric and psychological disturbances associated with the active hypercortisolemic state include mood disorders, particularly major depression, but also mania, anxiety disorders, psychological symptoms, and cognitive impairment, and it has been found that quality of life may be seriously impaired during both active and post-treatment phases [3]. Patients have also reported being particularly bothered by fatigue/ weakness and changes in physical appearance, as well as interference with family life and relations with their partner and impaired school or work performance [4]. Studies have shown that even patients who have been cured of the disease score lower in terms of general well-being, anxiety and depression, and overall quality of life than healthy controls [5]. For all of these reasons, it has been suggested that assessment of health-related quality of life (HRQOL) in Cushing’s patients is of prime importance and that it should complement existing clinical indicators of health status [3, 6].

To date, several studies have evaluated HRQOL in patients with CS, though the majority have used generic HRQOL measures such as the SF-36 [7], the SF-12 [8] and/or measures of specific symptoms associated with the disease, such as the Hospital Anxiety and Depression Scale [9]. It was only relative recently that a disease-specific measure (the CushingQOL) became available to measure HRQOL in patients with the condition [6, 10–12].

In addition to simply assessing HRQOL in patients with CS, it can also be important to obtain social preferences (or health utilities) for the disease states associated with the disease. Health utilities are of particular importance in economic evaluations of health-care technologies and interventions. Instruments used to collect and provide health utilities include the EuroQol 5D (EQ-5D) [13], the SF-6D [14, 15], and the Health Utilities Index [16]. In cases where this type of preference-based measure was not included in the initial data collection, but a disease-specific measure was, it may be possible to create a preference function which allows scores on the disease-specific measure to be ‘mapped’ to index values on the preference-based instrument. If mapping is successful, this approach can allow cost-utility measurements to be carried out even when a preference-based instrument was not used in the initial evaluation of an intervention [17].

Currently, health utilities for the EQ-5D are not available for Cushing’s syndrome patients. The aim of the present study was therefore to construct a prediction model to obtain EQ-5D health utilities for CS using scores on a disease-specific HRQOL measure (CushingQOL).

## Methods

### Study sample and data collection

Data used in the present analysis were from the European Registry on CS (ERCUSYN) [12]. ERCUSYN is a web-based, multicenter, observational study that enrolled CS patients from 36 centers in 23 European countries diagnosed after January 1, 2000, to October 31, 2010. The study used a mixture of prospective and retrospective recruitment, though primarily prospective. Patients were classified in the following four major groups depending on the diagnosis: pituitary-dependent CS (PIT-CS), adrenal-dependent CS (ADR-CS), CS from an ectopic source (ECT-CS), and CS from other etiologies (OTH-CS). Etiologic classification was based on histologic documentation of ACTH-secreting or adrenal tumor or biochemical and clinical resolution of hypercortisolism after surgical resection if histological reports were not available. Patients with adrenal cancer were excluded from the database. For the purposes of the mapping exercise, only the PIT-CS and ADR-CS patients were used.

Data collected in the ERCUSYN database included information on patients at diagnosis, such as baseline demographic and anthropometric characteristics, etiology of CS and diagnosis date, comorbidities, and bone status, among others. HRQOL was measured using the CushingQOL and the EQ-5D. Detailed data were also collected on CS therapy, and the long-term outcomes of treatment were assessed based on biochemistry and imaging parameters, post-treatment hormone replacement therapies, pituitary deficiencies, clinical features, QOL, and bone status. Urine 24-h free cortisol (UFC) levels were also collected in each visit and were assessed by physicians to determine whether they were within the range of normal values.

The ERCUSYN study was approved by the ethics committee of the Hospital Sant Pau, Barcelona, Spain, which was the coordinating center for the project. Local ethics committee for each participating institution also approved the study, and all patients provided written or verbal informed consent to participate, depending on national legal requirements.

As mentioned, HRQOL in the ERCUSYN study was measured using the CushingQOL [6] and the EQ-5D [13].

#### CushingQOL

The CushingQOL is a disease-specific questionnaire designed to assess HRQOL in CS. It is a self-reported instrument consisting of 12 questions which cover the areas of trouble sleeping, wound healing/bruising, irritability/mood swings/anger, self-confidence, physical changes, ability to participate in activities, interactions with friends and family, memory issues, and future health concerns. Content for the questionnaire was derived from interviews with 10 patients with the condition [10]. Patients respond on Likert scales with five response categories (‘Always,’ ‘Often,’ ‘Sometimes,’ ‘Rarely,’ and ‘Never,’ or ‘Very much,’ ‘Quite a bit,’ ‘Somewhat,’ ‘Very little,’ and ‘Not at all’). Responses are scored on a scale of 1–5, where ‘1’ corresponds to ‘Always’ or ‘Very much’ and ‘5’ to ‘Never’ or ‘Not at all.’ The overall score is calculated by summing responses on all items and ranges from 12 (worst HRQOL) to 60 (best HRQOL). To facilitate the interpretation of scores, they can be standardized on a scale from 0 (worst HRQOL) to 100 (best HRQOL).

#### EQ-5D

The EQ-5D is a generic, preference-based instrument which measures health status in 5 dimensions: mobility, self-care, usual activities, pain and discomfort, and anxiety and depression [18]. Each dimension has three response levels: no problems, some problems and either extreme problems (in the case of the pain/discomfort and anxiety/depression dimensions), unable to (in the case of self-care and usual activities) or confined to bed (in the case of mobility). Utility indices for the EQ-5D are available for several countries and provide weights on a scale anchored at 0 (dead) to 1 (full health) for each of the 243 states defined by the descriptive system. These are used for the estimation of QALYs, based on the stated preferences of members of the general public [19]. Respondents are also asked to rate their overall health status on a 0–100 hash-marked, vertical visual analog scale (EQ-VAS) on which 0 represents the worst imaginable health state and 100 represents the best possible imaginable health state. For the present analysis, health utilities derived from the UK value set were used [20], and derived using the following formula:

$$ \begin{aligned} {\text{Utility value}} = & 1- 0.0 8 1\, { }\left( {\text{if at least one 2 or 3}} \right) - 0. 2 6 9\, { }\left( {\text{if at least one 3}} \right) \\ & - 0.0 6 9\, { }\left( {{\text{if mobility}} = 2} \right) - 0. 3 1 4\, { }\left( {{\text{if mobility}} = 3} \right) \\ & - 0. 10 4\, { }\left( {{\text{if self care}} = 2} \right) - 0. 2 1 4\, { }\left( {{\text{if self care}} = 3} \right) \\ & - 0.0 3 6\, { }\left( {{\text{if usual activities}} = 2} \right) - 0.0 9 4\, { }\left( {{\text{if usual activities}} = 3} \right) \\ & - 0. 1 2 3\, { }\left( {{\text{if pain/discomfort}} = 2} \right) - 0. 3 8 6\, { }\left( {{\text{if pain/discomfort}} = 3} \right) \\ & - 0.0 7 1\, { }\left( {{\text{if anxiety/depression}} = 2} \right) - 0. 2 3 6\, { }\left( {{\text{if anxiety/depression}} = 3} \right). \\ \end{aligned} $$

For full health (11111), a utility value of 1 is assigned.

#### Other variables

Socio-demographic data (age, gender, level of education, employment status) and the following clinical variables were collected: blood pressure, date of diagnosis of CS, clinical type (pituitary or adrenal adenoma), UFC levels, presence of symptoms (muscle weakness, loss of libido, hair loss, menstrual irregularity, hirsutism, etc.), use of pharmacological treatment, prior surgical intervention for the disease, and comorbidities. UFC levels were reported for each follow-up visit. As the normality of these values was not assessed by a centralized laboratory, physicians were asked to classify them as (1) ‘against diagnosis’ (i.e., normal values), or (2) ‘supporting diagnosis’ (high or abnormal values).

### Model development and selection

Regression analysis was used to analyze the relationship between the EQ-5D utility score and scores on individual items in the CushingQOL. In all models, the dependent variable was the EQ-5D utility score. Models were additive Generalized Linear Models incorporating main effects. Several different models were tested to determine which was the best, based on criteria described below. The models included clinical and socio-demographic variables as well as individual CushingQOL items and different categorizations of CushingQOL scores as independent variables, transformations (logarithm or square root) of scores, interactions, and/or quadratic terms as predictors. Tobit models were also tested using a value of ‘1’ (perfect health on the EuroQol index) as the left-censored value.

Clinical and socio-demographic variables were initially tested for potential inclusion in the models by determining whether they showed a statistically significant association with EQ-5D utility scores. Categorical variables were analyzed using analysis of variance and continuous variables using Pearson’s correlation coefficient.

With regard to the CushingQOL itself, and the overall score, each item was tested individually in the models, by including them as discrete dummy variables (always vs. other response options). Items included were those which were significant at *p* < 0.01 in bivariate analysis. We also tested the following categorizations of CushingQOL scores by including them as dummy variables: presence of ‘1’ in any of the items answered; presence of ‘5’ in any of the items answered; overall score ≤ 20, between 21 and 40, between 41 and 60, between 61 and 80, and >80.

Analyses were performed using SAS^®^ (PROC REG and PROC GLM for ORL models). The following four criteria were used to select the final model: the model’s explanatory power (assessed using adjusted R-squared); the consistency of the estimated coefficients (sign and parameter estimation); normality of prediction errors; and simplicity. The normality of the prediction errors was assessed using mean error (ME), mean absolute error (MAE), root mean squared error (RMSE), and a percentage error under 5, 10, and 15 % of the overall scale of independent variable. The model’s simplicity was evaluated by determining whether predictors were readily available and how many predictor variables the model required. The criterion of simplicity was important in order to optimize model usability. In general in this type of modelling exercise, simple additive models performed almost as well as more complex models providing little extra advantage [17].

## Results

A total sample of 511 patients with PIT-CS or ADR-CS diagnosis was included in ERCUSYN registry. A final sample of 129 evaluable patients (98 with PIT-CS and 31 with ADR-CS) was included in the analysis, and 382 patients were excluded because of missing data on either the EQ-5D or the CushingQOL. Table 1 shows the basic socio-demographic and clinical characteristics of the mapping sample. Patients from 17 countries were included (Austria, Belgium, Bulgaria, Czech Republic, England, Estonia, France, Germany, Hungary, Italy, Latvia, The Netherlands, Norway, Portugal, Spain, Sweden, and Turkey). France included the highest number of patients (*n* = 35) and England, Germany, and Norway the fewest (*n* = 1).

**Table 1:** Socio-demographic and clinical characteristics of the original validation study sample in ERCUSYN database

Characteristic	*N* = 129
Mean (SD) age in years	43.3 (13.1)
Sex, female, *n* (%)	109 (84.5 %)
Education, secondary or university studies, *n* (%)	92 (71.3 %)
Time since diagnosis in months, mean (SD)	7.0 (9.8)
Clinical type, *n* (%)	
Adrenal adenoma	31 (24.0 %)
Pituitary dependent	98 (76.0 %)
UFC (nmol/24 h), mean (SD)	1023.4 (1051.7)
UFC interpretation, *n* (%)	
Supporting diagnosis	107 (83.0 %)
Against diagnosis	8 (6.2 %)
Not available	14 (10.8 %)
Receiving pharmacological treatment for CS, *n* (%)	28 (22 %)
Prior surgery, *n* (%)	77 (59.7 %)
Concomitant morbidities, *n* (%)	
Weight gain	107 (83 %)
Hypertension	94 (72.9 %)
Cushing’s skin symptoms	102 (79.1 %)
Diagnosed fractures	24 (18.6 %)
Depression	46 (35.7 %)
Diabetes mellitus	35 (27.1 %)
Muscle weakness	91 (70.5 %)
Loss of libido	31 (24.0 %)
Hair loss	34 (26.4 %)
Hirsutism	54 (41.9 %)
Irregular periods	57 (44.2 %)
CushingQOL score	
Mean (SD)	39.7 (17.1)
Median (IQR)	39.6 (25)
EQ-5D utility values	
Mean (SD)	0.55 (0.30)
Median (IQR)	0.62 (0.21)

Table 2 shows the score distributions on the CushingQOL and EQ-5D. The mean (SD) score on the CushingQOL was 39.7 (17.1). The EQ-5D showed a mean (SD) index score of 0.55 (0.3) on a scale of −0.59 to 1, and minimum and maximum scores of −0.32 and 1.

**Table 2:** Score distributions on the CushingQOL and EQ-5D

Values	Mean	Theoretical range	Standard deviation	Minimum	Median	Maximum	Valid *N*
CushingQOL	39.71	0–100	17.05	0	39.58	83.33	129
EQ-5D utilities	0.550	−0.594 to 1	0.300	−0.319	0.620	1	129

**Fig. 1:**
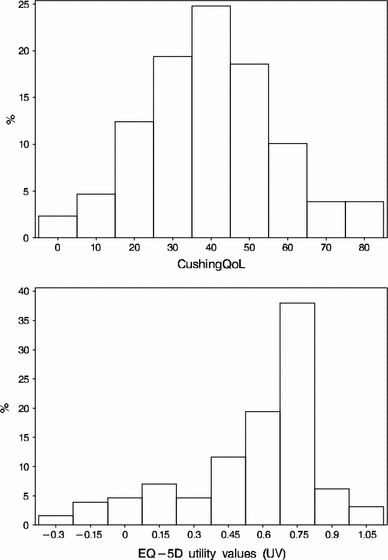
Distributions of CushingQOL scores and EQ-5D utility values in the simplest, reference model

Figure 1 shows the relative distribution of CushingQOL and EQ-5D total scores in the basic, two variable regression models. While it can be seen that the CushingQOL scores follow a relatively normal distribution, this is not true for EQ-5D index scores, which are skewed substantially to the right, that is, toward better HRQOL.

**Fig. 2:**
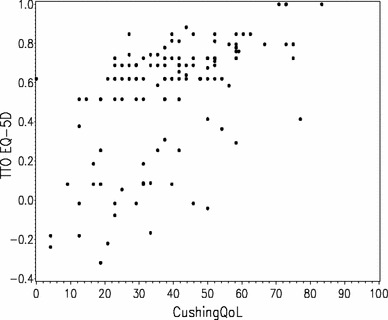
Correlation between CushingQOL scores and EQ-5D utility values in the reference model

Figure 2 shows the results of regressing the EQ-5D overall scores on CushingQOL overall scores. Higher EQ-5D index scores correspond to higher CushingQOL scores, though approximately 15 % of the CushingQOL scores recorded corresponded to EQ-5D values at or under 0.1, which represent extremely poor health states; 9.3 % had utility scores under 0, representing health states worse than death. Based on the slope, a 10 unit increase in the CushingQOL would be equivalent to an increase of almost 0.1 on the utility scale. The correlation between the two scores was 0.604.

Table 3 shows the results of testing the most promising models. In bivariate analyses, variables showing a statistically significant (*p* < 0.05) correlation with EQ-5D utility values were questions 2, 4, 5, and 10, and these can be considered to constitute the final model which we recommend for utility prediction with EQ-5D. Although having a diagnosis of clinical depression and employment status was also significant in the model, they were not included in order to simplify the model and because the relevance of employment status within the model was not clear. None of the other socio-demographic and clinical variables tested showed a statistically significant relationship with EQ-5D utility scores. The model which best met the criteria of explanatory power, consistency of estimated coefficients, normality of prediction errors, and simplicity was therefore Model 1. This model incorporated only 3 dummy variables, which take into account the impact of CushingQOL questions 2, 5, and 10. Although Models 2 and 4 showed a slightly better adjusted R2 than Model 1, the difference was minimal and came at the expense of less simple models. Utility values derived from model 1 can be obtained applying the following formula:

$$ \begin{aligned} {\text{Utility value}} = & 0. 8 3 5- 0. 4 8 5 { }\left( {\text{if level 1 in Q\hbox{-}02}} \right) - 0. 2 1 4\, { }\left( {\text{if level 2 in Q\hbox{-}02}} \right) - 0. 1 5 4\, { }\left( {\text{if level 3 in Q\hbox{-}02}} \right) \\ & - 0. 1 3 9\, { }\left( {\text{if level 1 or 2 in Q\hbox{-}05}} \right) - 0. 2 1 9\, { }\left( {\text{if level 1 in Q\hbox{-}10}} \right). \\ \end{aligned} $$

**Table 3:** Comparison of results obtained with the most promising models and the reference model (Model 1)

Parameter	Model 1	Model 2	Model 3	Model 4
N obs used	**128**	128	126	128
Intercept	**0.835**	0.844	−1.392	0.245
Level 1 in Q-02	−**0.485**	−0.470	0.920	0.360
Level 2 in Q-02	−**0.214**	−0.207	0.514	0.185
Level 3 in Q-02	−**0.154**	−0.140	0.384	0.170
Level 4 in Q-02	−**0.086 (ns)**	−0.092 (ns)	0.064 (ns)	0.097
Level 1 in Q-04				0.111 (ns)
Level 2 in Q-04				0.200
Level 3 in Q-04				0.181
Level 4 in Q-04				0.034 (ns)
Level 1 in Q-05	**−0.139**	−0.183	0.321	
Level 2 in Q-05	**−0.139**	−0.110 (ns)		
Level 3 in Q-05		−0.011 (ns)		
Level 4 in Q-05		0.073 (ns)		
Presence of “1” or “2” in Q-05				0.116
Level 1 in Q-10	**−0.219**	−0.246	0.306	0.157
Level 2 in Q-10		−0.046 (ns)		
Level 3 in Q-10		−0.033 (ns)		
Level 4 in Q-10		−0.048 (ns)		
*Model fit statistics*				
R^2^	0.5060	0.5196	0.4285*	0.5619*
Adj R^2^	0.4815	0.4695	0.3992*	0.5244*
RMSE	0.2163	0.2188	0.4423*	0.1552*
			0.2280	0.2025
MAE	0.160	0.158	0.161	0.153

*Responses* Level 1 (always), level 2 (often), level 3 (sometimes), and level 4 (rarely). Level 5 (never) is reference category

Values indicated in bold denote that the model 1 was chosen as the better one

MAE, mean absolute error; RMSE, root mean square error; Q-02, I have pain that keeps me from leading a normal life; Q-04, I bruise easily; Q-05, I am more irritable, I have sudden mood swings and angry outbursts; Q-10, My illness affects my everyday activities such as working or studying; Model 1 and model 2, ORL using utility values (UV) without transformations; Model 3, ORL using as response log((−1 × UV) + 1); Model 4, ORL using as response sqrt((−1 × UV) + 1)

* Values obtained directly from the model, prior to un-transforming to original utility values

Table 4 shows the results of analyzing the residuals in the selected models. Differences between observed and estimated mean and median values were generally small in all models. Error terms were obviously larger for maximum and minimum values because of much smaller number of patients scoring at the extremes. Although none of the prediction errors showed a normal distribution (Kolmogorov–Smirnov and Shapiro–Wilks *p* = 0.02), visual inspection showed their distribution to be reasonably close to normal.

**Table 4:** Analysis of residuals in the most promising models

	Observed EQ-5D score	Predicted EQ-5D score	Error term	Absolute error term	Square error
*Model 1*					
Mean	0.548	0.548	0	0.160	0.044
SD	0.300	0.214	0.211	0.137	0.070
Minimum	−0.319	−0.008	−0.599	0.001	0.000
Q1	0.516	0.462	−0.129	0.044	0.002
Median	0.620	0.610	0.030	0.138	0.019
Q3	0.725	0.696	0.147	0.223	0.050
Maximum	1.000	0.835	0.628	0.628	0.395
*N*	128^a^	128	128	128	128
*Model 2*					
Mean	0.548	0.548	0	0.158	0.043
SD	0.300	0.217	0.208	0.135	0.071
Minimum	−0.319	−0.055	−0.566	0.001	0.000
Q1	0.516	0.448	−0.124	0.052	0.003
Median	0.620	0.581	0.030	0.131	0.017
Q3	0.725	0.705	0.131	0.212	0.045
Maximum	1.000	0.917	0.675	0.675	0.456
*N*	128	128	128	128	128
*Model 3*					
Mean	0.551	0.583	0.032	0.161	0.052
SD	0.300	0.186	0.226	0.161	0.098
Minimum	−0.319	−0.143	−0.763	0.004	0.000
Q1	0.516	0.511	−0.098	0.049	0.002
Median	0.620	0.637	−0.014	0.098	0.010
Q3	0.725	0.738	0.118	0.250	0.062
Maximum	1.000	0.750	0.659	0.763	0.582
*N*	126^b^	126	126	126	126
*Model 4*					
Mean	0.548	0.570	0.022	0.153	0.041
SD	0.300	0.223	0.202	0.134	0.067
Minimum	−0.319	0.021	−0.599	0.001	0.000
Q1	0.516	0.447	−0.078	0.052	0.003
Median	0.62	0.627	−0.002	0.104	0.011
Q3	0.725	0.726	0.136	0.233	0.054
Maximum	1.000	0.940	0.584	0.599	0.359
*N*	128	128	128	128	128

Log(−1 + 1) = log(0) = NA

^a^128 observations used in this analysis because of a missing value in question 10

^b^Only 126 observations used in this model as the log transformation of 1 is not a valid operation

**Fig. 3:**
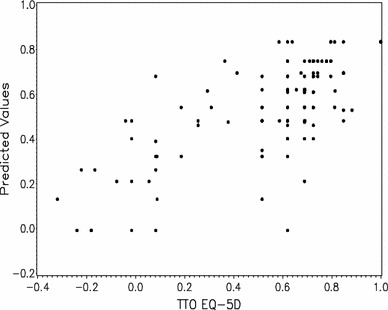
Predicted values and residuals using the final model

Figure 3 provides a graphic representation of predicted values and residuals using the final model. Higher values on the EQ-5D values are reflected in higher EQ-5D predicted values.

## Discussion

The EQ-5D is one of the most widely used measures of health status which also provides utility values for use in cost-effectiveness analyses. Having a mapping algorithm available to transform CushingQOL scores to EQ-5D utility values may therefore be very useful when it is not possible to obtain EQ-5D data directly from patients. In the present study, we derived a simple predictive model to map scores from the CushingQOL to the EQ-5D using data from the ERCUSYN study. The final model selected met our criteria for simplicity, consistency, and explanatory power, and prediction errors which did not deviate substantially from a normal distribution.

The final model selected included only 3 predictor variables, those being questions 2, 5, and 10 of the CushingQOL, though for the latter two questions only response levels 1 and 2 (representing the highest levels of severity) proved to be relevant for the model. These questions relate, respectively, to having pain that keeps the patient from leading a normal life, being more irritable or having sudden mood swings and angry outbursts, and the illness affecting everyday activities such as working or studying. These aspects of the CushingQOL clearly relate to individual dimensions on the EQ-5D which may explain why they were found to be the most relevant for the predictive model. Other questions in the CushingQOL may not have such a strong affinity with EQ-5D dimensions, which means they were not included in the model. However, despite the fact that conceptual overlap between the two instruments may not be high, the final model selected explained approximately 50 % of the variance in EQ-5D scores with a mean absolute error of 0.16. These results are similar to those observed in other studies which have mapped scores between different HRQOL instruments, including the EQ-5D [21–25]. Although other authors have questioned whether modeling should take place when there is a manifest lack of conceptual overlap between instruments [26], we consider the results of the present mapping exercise to be acceptable in terms of model performance parameters. It may be optimistic to expect greatly higher predictive capacity when mapping from disease specific to generic instruments. Likewise, although the model performs reasonably well on accepted performance parameters, it can clearly lead to predictive errors at the individual level that could be very high. For that reason, the model should only be used at the aggregate level, and not to estimate utilities for individual patients. Finally, it should be pointed out that modeling of this type is always a second-best choice to obtaining utilities by administering EQ-5D or other preference-based instruments directly in the population of interest.

A range of additional variables, as well as interaction terms, was tested to see whether they should be included in the models. However, only depression and employment status showed a statistically significant relationship with utilities and their inclusion in the models would help to improve the model’s adjustment. Nevertheless, it was decided not to include them in the models because data on the presence of clinical depression might not always be readily available and so would complicate application of the model, and because the conceptual relationship between employment status and utility values was insufficiently convincing to be included.

Alternative approaches to modeling were tested, such as using a Tobit model to take into account the skewed distribution on the EQ-5D, but found that it did not offer any advantages over the GLM models. In terms of usability, this is a relatively simple model, so it should not be too difficult to apply. However, users should be aware that the model is only relevant for the MVH UK tariff. Researchers wishing to transfer CushingQOL scores to other EQ country-specific tariffs would need to determine whether the same model can be applied, or whether they prefer to use the MVH UK tariff in other countries. It is also important to note the effects of the model when estimating values, such as the tendency to underestimate EQ-5D utilities across the measurement spectrum or to provide less reliable estimates at the extremes of the utility scale, especially in patients scoring below 0.2. If the utility values are to be used in economic modeling, these effects of the model could be taken into account to some extent in sensitivity analysis, by providing a range of possible EQ-5D utilities for specific categories of CushingQOL scores.

The ERCUSYN study represents the largest collaboration of endocrine centers in Europe and in the world in patients with CS [12]. Its strong points include the large sample size and the use of a standardized data collection protocol across all countries and centers. It is also the most recent large-scale study performed in CS patients, so the data should provide a very up-to-date picture of the impact of the disease and its treatment on health status and QOL. However, it was not the intention of the ERCUSYN registry to achieve a representative sample of CS patients in Europe. Clearly, this would have been preferable for mapping purposes. A noteworthy finding of the ERCUSYN study was the low EQ-5D index score (0.55). This was similar to or lower than index scores observed in patients with chronic heart failure [27] or COPD [28] and only a little higher than scores in patients with clinical depression [29]. Also of note was the fact that approximately 15 % of the subjects rated their own health on the EQ-5D as equal to or lower than 0.1, which represent a very low score indeed, and some of the patients even had ratings which would be equivalent to health states worse than death. This indicates the substantial impact of CS, though it should be noted that the scores on the CushingQOL observed here were considerably lower than the mean (SD) score of 53 (22) observed in the original validation study of the instrument [6], suggesting that the patients in the ERUSCYN study had considerably worse disease-specific health status than those in the earlier study. In fact, in the original validation study, 85 % had undergone surgery (more than the 59.7 % reported here) and only 31 % were hypercortisolemic compared to 83 % reported here. Finally, although a total of 511 patients were included for the final analysis in the ERCUSYN study itself, for the mapping exercise, only data from 129 patients could be used. Quality of life data were not collected from all patients because the study was carried out in conditions of usual clinical practice, and it was not always feasible for investigators to administer both of the HRQOL questionnaires.

### Study limitations

The fact that so many patients had to be excluded from the mapping analysis due to missing data on the EQ-5D and/or the CushingQOL questionnaire is an obvious limitation of the present exercise, as it lowered the sample size available and also threatened the sample makeup. Although the ERCUSYN study did not aim to provide a representative sample of CS patients, the reduction from 481 to 129 patients clearly leads to concerns about whether the patients used in the present exercise were even representative of the ERCUSYN sample or whether they could have represented an anomalous group. For that reason, we compared characteristics between the sub-group and the full ERCUSYN population to determine the extent to which the sub-group used in the present analysis was representative of the full ERCUSYN sample. Statistically significant differences were not found between groups on any of the major socio-demographic and clinical variables except employment status, in which there were proportionally fewer retired subjects in the sub-group analyzed here.

The limited sample size did not allow to test whether the model worked equally well in sub-samples of the overall population, for example, in PIT-CS and ADR-CS, or across different countries. Sample size calculations indicated that for six independent variables in the final model, we would require a minimum of 126 patients [30]. It would be interesting to evaluate this further in future studies, though as CS is a rare disease, it will usually be difficult to achieve sufficiently large samples. Regarding the issue of countries, as we only used the UK tariff of social values to obtain EQ-5D index scores, these values may not reflect the values of subjects in other countries. For example, some differences were found between the UK and Spain in terms of the weights assigned to different EQ-5D dimensions [31]. However, given the large number of countries involved and the relatively small number of patients in each country, using different country tariffs would not be practical and would likely have a negligible effect on results. Likewise, no country-specific tariff is available for many of the participating countries. In these situations, it is often recommended to apply the UK MVH tariff [19]. It should also be remembered that the model may had differed if, say, only patients from Spain or the UK had been included, as the relationship between CushingQOL and EQ-5D scores could have been affected. For example, if patients in the UK were, for cultural reasons, less willing to report anxiety and/or depression on the EQ-5D than their European counterparts, this would presumably lead to different coefficients for items related to that concept in the CushingQoL, particularly if they were less willing to report problems related to the concept on the CushingQOL as well. However, this is only speculation, as there was insufficient sample size to test this. Nevertheless, potential users of the model should be aware of. A final limitation was that a cross-validation test in another sample or in half of the original sample was not performed as the sample size was considered insufficient. It has been noted, though, that testing models in other samples do not often lead to any reduction in model performance [17].

In conclusion, the model developed here should be useful in transferring CushingQOL scores to the EQ-5D when the EQ-5D was not administered in the original study and when researchers are interested in using the UK tariff. The model should be easy to apply and showed acceptable goodness of fit. Future studies could examine whether the same model can be used with other country-specific utility tariffs for the EQ-5D.
